# Links Between Adiponectin and Dementia: From Risk Factors to Pathophysiology

**DOI:** 10.3389/fnagi.2019.00356

**Published:** 2020-01-08

**Authors:** RuiJuan Chen, Yi Shu, Yi Zeng

**Affiliations:** ^1^Department of Geriatrics, Second Xiangya Hospital, Central South University, Changsha, China; ^2^Department of Neurology, Second Xiangya Hospital, Central South University, Changsha, China

**Keywords:** adiponectin, dementia, vascular dementia, Alzheimer’s disease, risk factors, pathophysiology

## Abstract

With the aging population, dementia is becoming one of the most serious and troublesome global public health issues. Numerous studies have been seeking for effective strategies to delay or block its progression, but with little success. In recent years, adiponectin (APN) as one of the most abundant and multifunctional adipocytokines related to anti-inflammation, regulating glycogen metabolism and inhibiting insulin resistance (IR) and anti-atherosclerosis, has attracted widespread attention. In this article, we summarize recent studies that have contributed to a better understanding of the extent to which APN influences the risks of developing dementia as well as its pathophysiological progression. In addition, some controversial results interlinked with its effects on cognitive dysfunction diseases will be critically discussed. Ultimately, we aim to gain a novel insight into the pleiotropic effects of APN levels in circulation and suggest potential therapeutic target and future research strategies.

## Introduction

With the aging population, over 43.8 million people worldwide are suffering from dementia (GBD 2016 Dementia Collaborators, 2019). According to epidemiological statistics and estimates, by 2050, the population with dementia is predicted to triple worldwide (GBD 2016 Disease and Injury Incidence and Prevalence Collaborators, 2017). Furthermore among all chronic diseases, dementia is one of major causes leading to disability and dependance. Dementia will be one of the most serious and troublesome global public health issues (Alzheimer’s Association, 2013; GBD 2016 Disease and Injury Incidence and Prevalence Collaborators, 2017). The two most common forms of dementia are Alzheimer’s disease (AD) and vascular dementia (VD), accounting for 60% and 30% of dementia, respectively (Kalaria et al., 2008; Crous-Bou et al., 2017). Now, due to the rapid aging of the global population and the prolonged life expectancy, dementia has become a focus issue attracting global attention. This disease imposes a considerable burden on individuals, their families, and society.

**Figure 1:**
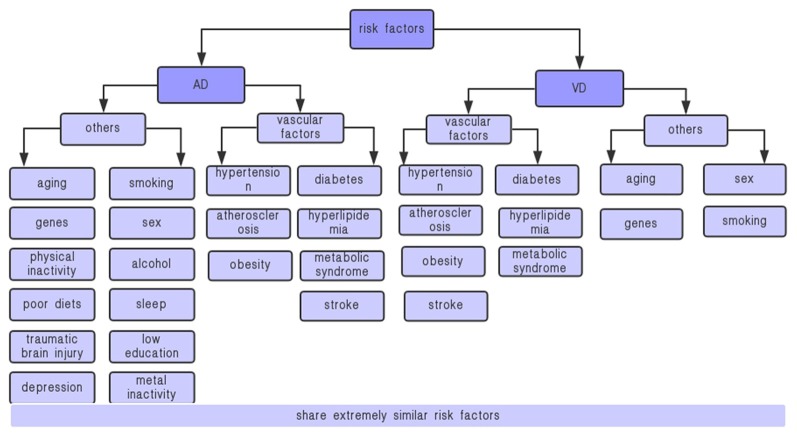
Risk factors for VD and AD. AD, Alzheimer’s disease; VD, vascular dementia. AD and VD share some extremely similar vascular factors, including hypertension, diabetes, metabolic syndrome, stroke, etc., which are closely related to adiponectin.

AD and VD are distinct diseases with potential overlapping metabolic dysfunction. They share some common risk factors, pathogenesis, and clinical features (Haan and Wallace, 2004). For instance, aging, sex, genetic factors, rate, and vascular factors, including hypertension, diabetes, dietary fat intake, metabolic syndrome (MetS), high cholesterol, stroke, and exercise are their common risk factors (Figure 1); peripheral and neuroinflammatory, IR, energy metabolism disorder, and oxidative stress are their common pathogenesis; cognitive and behavioral dysfunctions are their common clinical features.

APN is one of the most abundant adipocytokines. APN plays an important role in regulating vasodilation, resisting inflammation and anti-arterial atherosclerosis, increasing insulin sensitivity, and regulating glycogen and lipid metabolism. In this review, we summarize and discuss the latest advances in the association between APN and dementia, from the risk factors to potential pathophysiological mechanisms of dementia, hoping to explore a new way to address the pathophysiological mechanism associated with dementia.

## Adiponectin

Adiponectin (APN) is a fat-derived hormone that was first isolated from rat adipocytes by Scherer et al. (1995). Extensive attention was attracted as a key messenger for a connection between adipose tissue and other metabolically related organs. It is a 30-kDa protein, also known as ACRP30, apM1, adipoQ, or GBP28, containing 244 amino acids with an N-terminal collagen-like domain and a C-terminal complement factor C1q-like globular domain (Turer and Scherer, 2012). There are three different complexes of APN in the blood circulation: hexamers, trimers, and high molecular form (HMW; Aso et al., 2006; Wang and Scherer, 2016). Different forms own different biological activities. In related diseases, HMW APN or HMW APN/total APN may be a more effective indicator of insulin sensitivity (Aso et al., 2006). It works by binding to three receptors: AdipoR1, AdipoR2, and T-Cadherin. The three receptors are commonly expressed throughout the body, including adipose tissue, skeletal muscle, liver, pancreas, heart, blood vessels, and endothelial cells (Kiliaan et al., 2014). APN receptors have also been found in hippocampus, hypothalamus, and brainstem, and AdipoR1 expression is more pronounced in the brain (Letra et al., 2019b).

APN participates in regulating fatty acid and glucose catabolism and sensitizing cellular to insulin (Gustafson, 2010; van Himbergen et al., 2012; Letra et al., 2019b). APN has been repeatedly reported that it is negatively correlated with IR, body mass index (BMI), type 2 diabetes mellitus (T2DM), and cardiovascular disease. All these factors can elevate dementia risk (Wang and Scherer, 2016). In addition, APN exerts anti-inflammatory effects by decreasing the production of pro-inflammatory cytokines, including interleukin-6 (IL-6), interferon γ (INFγ) and tumor necrosis factor α (TNF-α), meanwhile increasing interleukin-1 (IL-1) and interleukin-10 (IL-10) expression (Brochu-Gaudreau et al., 2010). Furthermore, it reduces IR in the brain, but also in peripheral organs. Moreover, the role of APN in dementia with neurodegenerative symptoms has been confirmed in many studies.

## Dementia

Dementia delineates a clinical syndrome that is characterized by a series of memory difficulties, language disorders, psychological and mental changes, and impaired activities of daily living (Burns and Iliffe, 2009). AD is an irreversible and disabling progressive neurodegenerative disease owing to neuronal and synaptic loss. It is sporadic or shows modest familial clustering. AD tends to be more insidious than VD. It is similar to the symptoms of “normal aging” memory loss or communication disorders, and personality or emotional changes in the early states (Robinson et al., 2015). The diagnostic criteria for AD have been recognized extensively. Hallmarks of neuropathology in AD include deposits of amyloid-β (Aβ) peptide extracellularly and accumulation of hyperphosphorylated *tau* (p-*tau*) in neurofibrillary tangles (NFTs; Dubois et al., 2016; Crous-Bou et al., 2017). Therefore, this provides detailed information on the molecular pathogenesis of AD. Then, recently, a great deal of effort have been undertaken targeting these pathological mechanisms in an attempt to find potential therapeutic point for AD, especially focusing on the metabolism of the Aβ peptide (Citron, 2010; De Strooper et al., 2010).

Although second only to AD, VD remains an important form of dementia plaguing the elderly population. It is a progressive disease caused by a decreased blood flow into the brain that affects cognitive ability as well as executive function. It is the most severe form of vascular cognitive dysfunction (VCI; Gorelick et al., 2011), which is defined as cognitive function alterations caused by various vascular factors (Hachinski and Bowler, 1993). According to the contribution of vascular pathology to dementia, VD is classified as different subtypes, including cortical VD, subcortical VD, strategic infarct dementia, hypoperfusion dementia, hemorrhagic dementia, hereditary VD (e.g., CADASIL), and AD with cardiovascular disease (O’Brien and Thomas, 2015). However, the most common causes of vascular brain injury or infarction are atherosclerosis and cardiogenic embolism, while the cognitive impairment caused by microinfarcts in the cortex and subcortex is mostly attributed to cerebral small vessel disease characterized by arteriosclerosis and lacunar infarction (O’Brien and Thomas, 2015). Accurate diagnosis of VD relies on assessment of clinical symptoms, neuropsychological measurements, and neuroimaging and final pathological confirmation (Kalaria, 2016). Numerous cerebrovascular disease (CVD) causes stroke injury and other tissue perfusion changes, along with neurocognitive disorders, behavioral symptoms, motor abnormalities, and autonomic dysfunction (Kalaria, 2018).

AD, VD, or CVD has common risk factors including obesity, hypertension, diabetes, IR, hyperlipidemia, and hyperhomocystinemia (Fillit et al., 2008; Craft, 2009; Purnell et al., 2009). Therefore, learning more about the risk factors and the mechanism factors contributing to dementia could promote new ways to prevent, improve, or delay the progression of dementia.

## APN and Multiple Risk Factors of Dementia

Due to a tremendous increase in dementia and the lack of effective treatment, it is urgent to identify all the risk factors that accelerate or inhibit the cognitive impairment process. In many previous studies, several important risk factors were analyzed, including hypertension, diabetes mellitus, dyslipidemia and obesity at midlife, atherosclerosis, stroke, genes, and other factors (aging, low levels of education, smoking, depression and physical inactivity, etc.; Baumgart et al., 2015). AD and VD share the common risk factors especially vascular risk factors (Gardener et al., 2015). These common vascular risk factors indicate a correlation and potential interaction between AD and vascular pathology; however, the deep mechanism is unclear.

Some studies have suggested that the clinical manifestations of dementia are serious when vascular diseases co-exist (Snowdon et al., 1997; Jellinger and Attems, 2015). Identifying and reducing risk factors are major strategies for primary prevention. Here, we review the current research progresses on the dementia risk factors, as summarized in Figure 1 (Kalaria et al., 2008; Song et al., 2014; O’Brien and Thomas, 2015; Crous-Bou et al., 2017).

### Aging

Aging is interrelated with progressive declines in physiological function, which leads to multiple chronic diseases and frailty (Kirkland, 2013). Undoubtedly, dementia is an age-related disease. Some epidemiological studies have shown that between 65 and 90 years old, even in people aged 90 years and older, the incidence of all-cause dementia increases exponentially with age and doubles every 5 years (Jorm and Jolley, 1998; Corrada et al., 2010), Systemic decline with aging is characterized by various alterations, including mitochondrial dysfunction, cellular senescence, metabolic declines, adipose tissue dysfunction, IR, chronic sterile inflammation, and dysregulated nutrient sensing (Stout et al., 2017). Notably, IR and chronic low-grade inflammation are two major features of aging that occur nearly in most age-related diseases, such as dementia, diabetes, arthritis, CVD, and cancer. Besides, along with aging, adipose tissues undergo significant changes in distribution, abundance, endocrine signaling, and cellular composition. They play a central role in the development of IR, inflammation, metabolic dysfunction, and regenerative capacity (Palmer and Kirkland, 2016). As one of the most abundant adipocytokines secreted by adipose tissue, APN has versatile properties to positively influence these fundamental aging mechanisms. It is well known as a modulator in improvement of insulin sensitivity, inhibition of systemic inflammation, regulation of lipid and glycogen metabolism, and reduction of atherosclerotic processes (Brochu-Gaudreau et al., 2010). Numerous epidemiologic researches have indicated that low-circulating levels of APN are associated with many age-related metabolic disorders including T2DM, obesity, and cardiovascular disease (Arita et al., 1999; Lindsay et al., 2002, 2003). Besides, macrophage is crucial to regulate the aging process and prominently contribute to inflammatory and immune responses, but also helps maintain metabolic homeostasis (Biswas and Mantovani, 2012). It is reported that APN expressed in macrophages improves insulin sensitivity and protects against inflammation and atherosclerosis (Luo et al., 2010). Reduced IR and inflammation appear to be closely associated with life span (Franceschi et al., 2005; Ewald et al., 2018). In addition, mitochondria impairment increases with age, leading to age-associated disease phenotypes and senescense (Kauppila et al., 2017). Iwabu et al. (2010) dedicated that decreased levels of APN and AdipoR1 in obesity may play causal roles in IR and mitochondrial dysfunction seen in diabetes. Several recent studies suggest that mitochondrial dysfunction is linked to impairment of insulin sensitivity and decreased APN secretion in adipocytes (Koh et al., 2007; Wang C. H. et al., 2013). Considering these evidences, APN might be a key factor in aging pathway.

### Hypertension

Emerging as a leading cause of age-related cognitive dysfunction, hypertension is known to be linked to VD, and is also associated with AD and other adverse cognitive outcomes (Crous-Bou et al., 2017; Iadecola and Gottesman, 2019). These evidences primarily come from many epidemiological studies, which strongly support the idea that hypertension is clearly correlated with steeper cognitive decline. But this adverse effect mainly occurs in middle age, early stage in dementia (Kennelly et al., 2009), while some studies show that later-life hypertension may help to prevent cognitive decline (Kennelly et al., 2009; Corrada et al., 2017). Deficit of nitric oxide (NO) derived from endothelial NO synthase (eNOS) and endothelial dysfunction is one of the vital mechanisms for hypertension to affect cerebral blood flow and lead to cognitive decline (Delles et al., 2004). Endothelial cells secrete a variety of vasodilators (e.g., NO, prostaglandins) and vasoconstrictors (e.g., endothelin ET, thromboxane A2), thereby regulating vasoconstriction and altering cerebral perfusion (Vanhoutte et al., 2017). Meanwhile, the endothelium is also essential to maintain the blood–brain barrier (BBB) for bidirectional molecular transmission between brain and other organs of the body (Sweeney et al., 2019) and exerts crucial trophic effects on brain cells (Marie et al., 2018). Previous studies demonstrated that hypertension causes severe damage to vascular endothelial cells, which in turn leads to BBB permeability changes, dysfunction of cerebral vascular and perivascular, and brain structural failure, including subclinical brain infarcts, white matter hyperintensities, and cerebral microbleeds (Iadecola and Gottesman, 2019). Furthermore, vascular injury and perivascular dysfunction caused by hypertension may impair the Aβ disposal and accumulation. Conversely, loss of NO bioavailability associated with vascular dysfunction may also contribute to the promotion of increased Aβ and amyloid formation, which is a marker in AD. APN decreases the risk of hypertension and improves cognitive impairment by stimulating NO production. It promotes NO release *via* AdipoR1 and AdipoR2 and inhibits cerebral inflammatory response through adenosine monophosphate-activated protein kinase (AMPK)/eNOS signaling pathway activation (Shibata et al., 2004). Increased NO reduces platelet aggregation and elevates vasodilation regulating cerebrovascular microcirculation. In addition, APN could suppress amyloid-β in mice (Jian et al., 2019). Waragai et al. (2016) have suggested a possible positive effect of APN by finding that higher cerebrospinal fluid (CSF) level of APN is related with lower amyloid and *tau* burden. Consequentially, APN could decrease the risk of hypertension and improve vascular cognitive impairment. Despite this, the interaction between APN and hypertension, one of the risk factors in dementia, still has many unsolved mysteries that require further reveals.

### Obesity

Obesity is another independent risk factor of dementia in the diverse adult urban population. Traditionally, obesity refers to an increase in whole body mass, which cannot analyze body composition or distinguish between subcutaneous and visceral fat, while excessive accumulation of visceral fat can better illustrate metabolic abnormalities (Duvnjak and Duvnjak, 2009).

Overweight and obesity are the cornerstones of vascular risk contributed to various diseases. BMI, waist circumference, and waist-to-hip ratio are widely quantitative measurement methods to assess obesity, and the BMI is the most effectively popularized in clinic (over 30 kg/m^2^). Significant increase in abdominal circumference means central adiposity or abdominal obesity, which is more associated with visceral fat deposits and has a stronger relationship with adverse metabolic outcomes (Luchsinger, 2008). Abdominal obesity, hypertension, dyslipidemia, and IR are collectively defined as MetS. MetS is a systemic inflammatory response, which is a cluster of risk factors interrelated with cardiovascular disease and T2DM (Grundy et al., 2005; O’Neill et al., 2016). Epidemiological evidence suggests that MetS may also be linked to cognitive dysfunction, involving VD and AD (Martins et al., 2006; Cooper et al., 2015). Obesity and MetS represent an increase in adipose tissue and lead to an adipocyte endocrine dysfunction, secreting excess or inadequate adipose tissue hormones and adipokines, which may be a clue to the mechanisms associated with dementia. APN regulates cerebral inflammatory responses, central food intake, energy expenditure glucose, and fatty acid catabolism. It is also a potent insulin sensitizer with a negative correlation with obesity, T2DM, MetS, and cardiovascular diseases (Chandran et al., 2003). Thus, it exerts beneficial effects on neuroprotection, neurotrophic actions, and neurogenesis. This may be attributed to modulation of insulin receptor signaling, sensitizing the insulin receptor signaling pathway and suppression of neuroinflammation. Though much data support obesity associated with multiple types of dementia, the links between adipokines and dementia risk remain to be further explored.

### Diabetes

T2DM is also linked with dementia. It is characterized by hyperglycemia, IR, and pancreatic β cell dysfunction. Epidemiology studies have shown that the probability of cognitive decline in elder patients with T2DM is 1.5 times higher than that of non-diabetics (Biessels et al., 2006; Cheng et al., 2012). The latest cross-sectional and longitudinal researches have strongly demonstrated their association (Cukierman et al., 2005; Biessels et al., 2006). In addition, recent studies suggest that AD is a brain-specific diabetes and define it as “type 3 diabetes” on an account of the common risk factors between diabetes and AD, such as IR, BBB disruption, and altered glucose homeostasis (de la Monte and Wands, 2008; Kroner, 2009; de la Monte, 2014). But the exact mechanism between T2DM-related dementia especially in AD is unclear. The ultimate underlying mechanism is possibly related to IR, and systemic inflammatory response is associated with diabetes, vascular abnormalities, neurodegenerative changes, and other multifactorial effects. Combined mechanisms may lead to mixed pathology. First, CVD pathology is likely to be an important determinant of the risks of all-cause dementia in individuals with diabetes. In several different studies, T2DM was consistently associated with an increased risk of pathologically verified infarcts at autopsy (Arvanitakis et al., 2006; Pruzin et al., 2017). These macroscopic brain infarcts may contribute to insidious ischemia of the brain-related impaired cognitive function (Pruzin et al., 2018). Another potential pathology is neurodegenerative change including deposits of neurotic NP and NFTs, which may be attributed to brain IR and altered insulin signaling (Verdile et al., 2015). In addition, disruption of the BBB, dysregulation of lymphatics, and activation of the hypothalamic–pituitary–adrenal (HPA) axis are also proposed in many published articles (Ng and Chan, 2017; Pruzin et al., 2018). In conclusion, present data indicate that the relationship between T2DM and dementia is probably multifactorial in etiology. APN plays an important anti-inflammatory and anti-oxidant role, enhancing insulin sensitization and maintaining BBB and anti-atherosclerotic properties, which are crucial potential mechanisms in diabetes-induced dementia as described above Ng and Chan (2017). Some studies have reported that subjects with T2DM have lower plasma APN concentrations than matched non-diabetic controls (Hotta et al., 2000), and low APN levels can be used as predictors of the incidence of T2DM (Lindsay et al., 2002; Spranger et al., 2003).

### Atherosclerosis

Atherosclerosis is a progressive vessel disease of large- or medium-sized arteries that eventually leads to cardiovascular diseases and stroke. Chronic inflammation, abnormal immune responses, and lipid depository are involved in the development of atherosclerosis (Solanki et al., 2018). Then, intracranial atherosclerosis (ICAS) causes degenerative vessel stenosis and cerebral hypoperfusion associated with an increased risk for ischemic stroke and dementia (Gorelick et al., 2008; Yarchoan et al., 2012). Atherosclerotic plaque development and rupture adding to subsequent rupture of thrombosis contribute to partial or total occlusion of the affected artery. These constitute main events of ICA (Wang et al., 2019). Based on valuable studies, APN may inhibit the release of pro-inflammatory cytokines (e.g., TGF-α), enhance the release of anti-inflammatory cytokines (e.g., IL-10), and promote the shift of macrophages toward the anti-inflammatory phenotype M2. Furthermore, it increases macrophages cholesterol efflux and prevent generation of foam cells (Lovren et al., 2010; Jenke et al., 2013; Wang M. et al., 2013). Another critical factor leading to atherosclerotic plaque is dyslipidemia, which is characterized by high triglycerides (TG), low high-density lipoprotein cholesterol (HDL-C), and small-dense low-density lipoprotein (sd-LDL) particles (Lusis, 2000). APN is well-known as a mediator in lipid metabolism. It is positively correlated with HDL-C and negatively associated with LDL cholesterol (LDL-C) and triglyceride concentrations (Katsiki et al., 2017). All evidences indicate that APN exerts a multifaceted effect in improving atherosclerosis in brain by regulating atherogenic factors.

### Stroke

Stroke is defined as a cerebral disease that the blood supply to the brain is interrupted and subsequently the brain lacks vital oxygen and nutrients, leading to focal or global disruption of neurological function, without other obvious cause apart from vascular disorders (WHO MONICA project principal investigators, 1988). Accumulating evidences suggest that vascular factors and stroke injury increase risk for dementia including AD, not only VD (Tosto et al., 2016; Nucera and Hachinski, 2018). Mechanisms might involve hypoxia, hypoperfusion, and neuroinflammation. Furthermore, dementia and stroke share the corresponding risks and protective factors (Nucera and Hachinski, 2018). Stroke increases the risk of dementia in two ways. One is a sharp decline in cognition that occurs after a stroke (post-stroke dementia), while the other is to accelerate the development of cognitive decline several years after stroke (Levine et al., 2015). Dementia becomes a severe problem in stroke survivors. Several prospective cohort studies have demonstrated cognitive deficits after stroke (Lusis, 2000; Katsiki et al., 2017). In addition, meta-analysis studies have shown that some form of cognitive impairment affects approximately 30% of stroke patients (Ben Assayag et al., 2012; Wollenweber et al., 2014). The risks for stroke are shared with dementia, including non-modifiable (e.g., age, sex, and genes) and modifiable (e.g., hypertension, diabetes, obesity, MetS, and smoking) factors (Vijayan and Reddy, 2016). Association between APN and stroke have been evaluated in a number of studies, but the results are contradictory. Some retrospective case–control studies suggest that low APN level is related to greater stroke risk (Stott et al., 2009; Savopoulos et al., 2011; Prugger et al., 2012). Furthermore, several studies suggest that APN knockout (APN-KO) mice are more prone to serious ischemia–reperfusion injury in the brain (Shibata et al., 2005). However, several cases report that there are no independent links between APN and stroke risk (Stott et al., 2009). In addition, some other studies have shown that elevated APN levels in the elderly may contribute to an increased risk of ischemic stroke (Hao et al., 2013).

Considering all above results, whether APN is positively correlated with the risk factors of stroke still requires a lot of research data to identify, though numerous studies have confirmed its affirmative effects on vascular risk factors such as anti-atherosclerosis, anti-inflammation, and IR.

### Other Risk Factors

Genetics, ethnicity, smoking, physical activity, social engagement, cognitive training, diet, traumatic brain injury, depression, sleep, and level of education, etc. These are also crucial factors influencing the progression of dementia. For instance, genetic diagnosis is becoming a hot spot in dementia research. But most of them have been on AD while investigations on VD have mainly been on rare familial syndromes. According to a system review involved of meta-analysis research, there are six polymorphisms strongly associated with vascular cognitive impairment (APOE, ACT, ACE, MTHFR, PON1, and PSEN-1 genes; Dwyer et al., 2013). APOE e4 has been reported as a significant related gene of AD in another independent meta-analysis (Farrer et al., 1997; Yin et al., 2012). Moreover, exercise is another important factor that has been reported to possibly maintain and improve cognitive function (Brown et al., 2013; Pedersen, 2019). A randomized controlled trial has shown that physical or mental activity may enhance cognitive function in older adults and that the amount of activity is more important than the type in the subject population (Barnes et al., 2013). Hence, distinguishing and utilizing these modifiable factors could help us find effective interventions to prevent dementia at the preclinical stage. Understanding well the role of APN in the progress is a key to explore novel strategies.

## Regulation Mechanism of APN on Dementia

**Figure 2:**
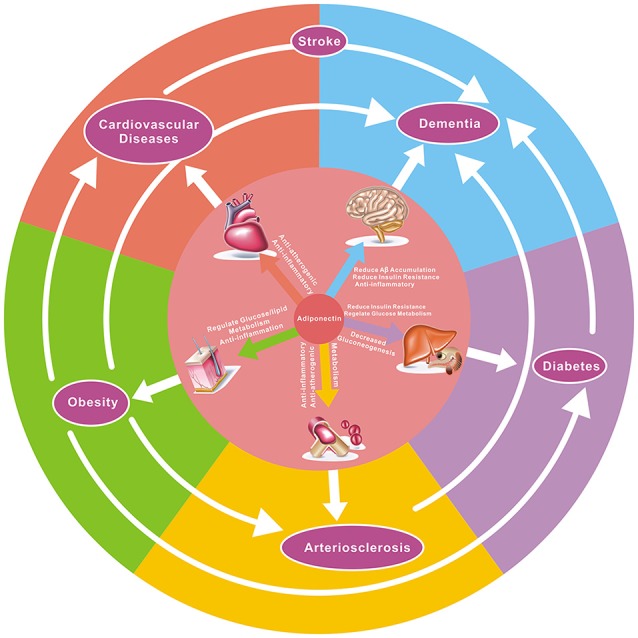
The potential effect of adiponectin to target diseases and the relationship between them. As demonstrated here, adiponectin has pleiotropic effects on numerous organs and tissues, and there is a close correlation between various diseases. For example, hypertension, atherosclerosis, diabetes, and obesity are risk factors in both dementia and coronary heart disease, while hypertension, atherosclerosis, and obesity are associated with the onset of diabetes. Adiponectin has anti-atherogenic, glucose metabolism-regulating, anti-inflammatory, insulin-sensitizing, and cardioprotective effects in multiple diseases.

APN has pleiotropic effects that may benefit AD and VD, involving anti-inflammatory and insulin-sensitizing effects, regulating sugar and lipid metabolism, regulating glucose and lipid metabolism, improving mitochondrial dysfunction, decreasing Aβ amyloid deposition, and inhibition of *tau* phosphorylation (Figure 2).

### Anti-neuroinflammation

Epidemiological and clinical studies have repeatedly demonstrated that APN plays a chronic anti-inflammatory role in dementia and other related diseases, such as T2DM, cardiovascular disease, and cancer. For example, Chabry et al. (2015) showed that APN could reduce neuroinflammation and depressive-like behaviors in mice by regulating microglia and macrophage phenotype and activation state. Lecompte et al. (2017) found that APN retained its anti-inflammatory feature in dystrophic muscle by activating the AdipoR1-AMPK-SIRT1-PGC-1αpathway in mice. Besides, Jian et al. (2019) demonstrated that APN suppressed inflammatory response of microglia to amyloid-β oligomer (AβO) and APN deficiency may aggravate microglia-mediated neuroinflammation in AD mice. In patients with severe carotid stenosis, APN’s negative association with glucose, insulin, and intraplaque inflammatory markers was observed (Liberale et al., 2018). Macrophages are the main targets for APN to exert anti-inflammatory effects. It acts by suppressing macrophage differentiation, modulating macrophage function or shifting phenotype from proinflammatory M1 state to an anti-inflammatory M2 state, decreasing expression of Toll-like receptor 4 (TLR4), and regulating inflammation responses (Yokota et al., 2000; Gordon and Martinez, 2010). It inhibits the transformation of human monocyte-derived macrophages into foam cells and the production of inflammatory chemokines and upregulates the production of the anti-inflammatory cytokine interleukin 10 (IL-10) in macrophages binding AdipoR1 primarily. Additionally, it may modulate the suppression of M1 macrophage activation; in contrast, it regulates promotion of M2 macrophage proliferation (Fang and Judd, 2018). It binds three receptor subtypes, AdipoR1, AdipoR2, and T-cadherin receptor, and mediates its effects primarily through AMPK signaling pathway (Thundyil et al., 2012). APN is also reported to act protective effects against inflammation on a variety of cell types (e.g., cardiomyocytes, endothelial cells, and vascular smooth muscle cells or microglia, astrocytes, and neurons in brain), and it mediates the phenotypes of these cells to exert anti-inflammatory effects (Ohashi et al., 2014; Sargolzaei et al., 2018). APN gene expression is widely expressed in the cortex and the hippocampus. It is suggested that APN inhibits pro-inflammatory signal release, IL-6 and TNF-α, from BBB endothelial cells, indirectly modulating the inflammatory signaling across the BBB (Spranger et al., 2006). In some studies, it is demonstrated that it down-regulates neuroinflammation by decreasing Aβ amyloid deposition in AD, while improving dementia (Chan et al., 2012; Song et al., 2017). Therefore APN’s anti-inflammation properties in target organs is multiplex and complicated, and worth discussing further.

### Inhibiting Insulin Resistance

Definition of IR in T2DM means “reduced sensitivity in body tissues to the action of insulin,” ordinarily observed in T2DM and obesity, characterized as hyperinsulinemia and dyslipidemia (Goldstein, 2002). Correspondingly, brain IR is equivalent to a weakened response of the brain cells to insulin (Mielke et al., 2005). Insulin is involved in neuromodulation, including regulation of neurotransmitter (e.g., acetylcholine) concentrations, neuronal differentiation, repair, proliferation, regeneration, and suppression of neuronal apoptosis, subsequently mediating memory and learning processes (Kang et al., 2017). Thus, brain IR may be attributed to a decrease in insulin receptors, a loss of insulin receptor-binding insulin function, or defective insulin signaling cascade in the central nervous system. At the cellular level, this lack of response may appear as alteration of neuroplasticity, receptor regulation or neurotransmitter release dysfunction, or insulin metabolism disorders. Functionally, dysregulation of brain or peripheral metabolism, as well as cognitive and emotional impairment might be the main manifestations. In addition, numerous epidemiological data propose that obesity, T2DM, and other MetS of IR are risk factors for AD and other associated dysfunctions (Ahtiluoto et al., 2010; Bosco et al., 2012; Gao et al., 2013; Chatterjee et al., 2016).

Besides, it is delineated that in IR, target tissues do not respond insufficiently to insulin stimulation, eventually leading to hyperglycemia, hyperlipidemia, inflammation, and reduced plasma APN concentrations (Shibata et al., 2005). Upregulating plasma APN can reverse the sequelae of IR in various target organs, such as skeletal muscle, liver, pancreatic, and adipose tissue, established by a mass of basic and clinical outcome (Combs et al., 2004; Otabe et al., 2007; Wang et al., 2014; Ye et al., 2014). In contrast, some other documents show that APN deficiency is closely related to IR-exacerbating metabolic disorders such as T2DM and obesity (Maeda et al., 2002; Spranger et al., 2003; Bajaj et al., 2004). In these diseases, infiltration of inflammatory cells, particularly activated macrophages, aggregating into adipose tissue induces a highly inflammatory status. Meanwhile, in these conditions, a significant reduction exists in serum APN (Zhang et al., 2009). In addition, studies have shown that continuing elevated levels of inflammatory cytokines may directly deteriorate IR and lead to disruption of insulin sensitivity (Weisberg et al., 2003; Xu et al., 2003). APN decreases oxidative stress and inflammatory cytokines, which contribute to an improvement of IR. Animals and human studies have also identified that serum APN is transported into the CSF, regulating various central physiological functions of the brain (Qi et al., 2004). Intraventricular injection of APN can improve hypothalamic insulin signaling activity in diabetic rats and adjust glucose homeostasis (Park et al., 2011). Therefore, APN defined as an insulin-sensitizing hormone is a crucial mediator participating in the protection of brain health. Exploiting and better understanding its function in the crucial pathological mechanism in nerve provide powerful opportunities to novel therapeutic interventions that may improve dementia.

### Regulating Glucose and Lipid Metabolism

As mentioned above, the molecular alterations in diabetes, obesity, and dementia are accompanied by impaired glucose uptake, lipid and fatty acid metabolism dysfunction, and energy metabolism disorder (Hotamisligil, 2006; Lourenco et al., 2015). Furthermore, increasing evidence has clearly demonstrated that cellular IR exists in the brains of AD patients and even those of non-diabetic patients; therefore, AD is also referred to as “type 3 diabetes” (de la Monte and Wands, 2008). This means that dementia especially AD can absolutely be considered a kind of metabolic disease (De Felice et al., 2014; Ferreira et al., 2014). However, APN directly or indirectly exerts anti-insulin resistance, regulation of glycogen, lipid and fatty acid metabolism, and reduction of oxidative stress at the hub or periphery. In terms of glycogen metabolism, it is involved in mediating hepatic glucose production, glycogen uptake, decomposition, utilization, storage, transport, energy expenditure, and protection of pancreatic β cell as well as maintaining glycogen homeostasis in the brain. While in lipid metabolism, it is demonstrated that circulating APN levels positively associate with HDL-C (Matsubara et al., 2002; Yamamoto et al., 2002; Ezenwaka et al., 2004; Kazumi et al., 2004; Shetty et al., 2004; Kangas-Kontio et al., 2010; Christou et al., 2012) and show an inverse correlation with TG (Matsubara et al., 2002; Yamamoto et al., 2002; Ezenwaka et al., 2004; Siebel et al., 2015; Yanai and Yoshida, 2019). The possible mechanisms underlying up-regulation of HDL-C due to APN involve increasing production of apolipoprotein apo-AI and ATP-binding cassette transporter A1 (ABCA1; Matsuura et al., 2007; Oku et al., 2007; Qiao et al., 2008; Kitajima et al., 2011), down-regulation of hepatic lipase (HL) activity (Schneider et al., 2005; Clarenbach et al., 2007), and activation of lipoprotein lipase (LPL; Yanai and Yoshida, 2019). On the other hand, the plausible mechanism of TG reduction can be attributed to the regulation of LPL activity by APN (von Eynatten et al., 2004; Kobayashi et al., 2005) and the decrease of APN-induced serum APO-CIII, a well-known LPL inhibitor (Chan et al., 2005; Tsubakio-Yamamoto et al., 2012). With regard to LDL-C, the majority of studies have indicated no association with circulating APN (Kazumi et al., 2004; Shetty et al., 2004; Tomono et al., 2018). Nevertheless, high sd-LDL levels have been demonstrated to be correlated with elevated TG levels and decreased HDL-C levels, which constitute a common feature of diabetes and MetS (Eckel et al., 2005; Rizzo et al., 2009). In addition, APN-mediated improvement of HDL and TG may decrease the atherogenic lipoprotein sd-LDL and remnant lipoproteins, derived from very low density lipoprotein (VLDL) and chylomicrons (Yanai and Yoshida, 2019). Summarily, increase of serum APN levels may protect against atherosclerosis and other related diseases *via* mediating lipid metabolism.

### Special Effects in AD Pathogenesis

AD is referred to a degenerative brain disease characterized by extracellular Aβ plaques and intraneuronal accumulation of NFTs. Amyloid plaques contain Aβ, while NFTs are composed of hyperphosphorylated *tau* proteins (Takahashi et al., 2017). APN has been repeatedly reported to play special effects in AD pathogenesis.

#### Reducing Aβ-Amyloid Deposition

Extracellular Aβ-amyloid deposition into oligomers, fibrils, and plaques is a major hallmark of AD pathological mechanism. It may cause the dysfunction of several crucial processes, such as synaptogenesis, neurotrophy, and apoptosis, showing neurotoxin in the disruption of learning and memory (Rad et al., 2018). Then, the amyloid cascade hypothesis is the most prevailing hypothesis and propose that Aβ accumulation is the initiating mechanistic event. In this case, various stages of aggregates, involving protofibrils of Aβ, fibrillar forms of Aβ, as well as different soluble and insoluble Aβ oligomers, are neurotoxic as they could damage synapses and, in turn, cause neuron loss, ultimately leading to chronic neurodegeneration and dementia (Hardy, 2009; Blennow et al., 2015). Currently, targeting this molecular mechanism of Aβ neurotoxicity, numerous research therapies emerge aiming to reduce further Aβ aggregation and plaque formation in brain, but remain invalid (Doody et al., 2013, 2014; Salloway et al., 2014). It could be that Aβ accumulation is just a bystander, rather than the cause, of neurodegeneration in AD. Studies have posed that insulin modulates various steps in the amyloid cascade, affecting Aβ aggregation in the brain. The disturbance of insulin signaling may inhibit Aβ clearance and accelerate the formation of neurotoxic Aβ plaque (Kim and Feldman, 2015; Rad et al., 2018). In addition, there is no doubt about the relevance of neuroinflammation in AD. Astroglia and microglia are believed to be the major sources of pro-inflammatory cytokines in the brain and can be stimulated by Aβ aggregation (Letra et al., 2019b). However, it has been reported that APN inhibits the inflammatory response and improve IR to indirectly reduce the production of amyloid plaques (Kamat et al., 2016). Furthermore, a study has shown that APN suppresses inflammatory response of microglia by inhibiting AβO and that APN deficiency promotes aggravation of microglia activation and deteriorates neuroinflammation in AD mice (Jian et al., 2019). Thus, it appears to be valuable to probe and perceive crosstalk of APN to AD for effective therapies.

#### Inhibiting Hyperphosphorylation of *Tau* in NFTs

NFTs are referred to as the twisted fibers involving abnormal phosphorylated *tau* proteins (also named phospho-*tau*, or p-*tau*), which exist as oligomers primarily in neurons suppressing microtubule assembly (Grundke-Iqbal et al., 1986; Iqbal et al., 1986). The lack of successful clinical trials targeting Aβ plaques provides a novel opportunity to seek out potential therapy targeting pathological *tau* in AD progression. The crucial role of *tau* has been emphasized by several clinical studies in regard to the close correlation between *tau*-positive NFTs and AD development in the brain. NFT-positive cell density correlates with disease stages, which are measured by clinical parameters for disease severity or cognitive decline (Giannakopoulos et al., 2003). Contrarily, senile plaque density is not associated with stages (Delaère et al., 1990). Hyperphosphorylated *tau* is neurotoxic, suppressing microtubule assembly and inducing prion-like template activity. Therefore, the most promising treatment is to inhibit hyperphosphorylation and clear pathological *tau*; in addition, nerve regeneration can save *tau* pathology and cognitive decline (Iqbal et al., 2016). Xu et al. (2018) found that in the ICV-STZ rat model experiment, APN supplements inhibit hyperphosphorylation of *tau* protein at multiple AD-related sites, improve cognitive deficits, and have neuroprotective effects. But intriguingly, it is also reported that adaptation of APN to IR may play a dual role in the formation of two markers of AD: Aβ plaques and NFTs, and perhaps its fluctuation acts as a driving force in the disease pathogenesis (Sekiyama et al., 2014; Waragai et al., 2016, 2017). Therefore, according to this unique biological mechanism of APN in AD, a selective therapeutic strategy that is distinct from previous concepts may be required.

## Controversy

Although a large number of studies have shown that APN has beneficial effects such as anti-inflammation, attenuating IR, regulation of sugar and lipid metabolism, and anti-atherosclerosis, its neuroprotective effects are still controversial. Some researches suggest that APN is of no significance, and some even think it is harmful (Table 1; Kamogawa et al., 2010; Une et al., 2011; Teixeira et al., 2013; Dukic et al., 2016; Gorska-Ciebiada et al., 2016; Kitagawa et al., 2016; Bednarska-Makaruk et al., 2017; Bossolasco et al., 2017; Fujita et al., 2018; Gilbert et al., 2018; Benavente et al., 2019; Letra et al., 2019a). Furthermore, high serum APN levels have been reported to be positively associated with cardiovascular mortality (Ortega Moreno et al., 2016). These conflicting results about association between the APN serum levels and the outcomes of different stages of disease (MCI or dementia) suggest that we still know very little about the complex involvement of APN in AD. What contributes to the incongruences described above? Here, are five possible underlying causes.

**Table 1:** Population-based studies aiming to assess the association between adiponectin and cognitive dysfunction diseases.

References	Study objects	Title	Disease	Sample, method	Results
Gorska-Ciebiada et al. (2016) and Xu et al. (2018)	62 seniors with type 2 diabetes (T2DM) and MCI, and 132 seniors with T2DM but without MCI (controls)	Adiponectin, leptin, and IL-1β in elderly diabetic patients with mild cognitive impairment.	Mild cognitive impairment.	Serum, ELISA	Serum leptin and IL-1β levels were higher and adiponectin concentration was lower in MCI patients than controls. In MCI subjects, adiponectin level was negatively correlated with leptin, IL-1β levels, and BMI. Leptin concentration was correlated with IL-1β level. Univariate logistic regression models revealed that the factors that increased the likelihood of diagnosis of MCI in elderly patients with T2DM were higher levels of HbA1c, leptin, IL-1β, and triglycerides, as well as lower levels of adiponectin and HDL cholesterol. Similarly, previous CVD, hypertension, hyperlipidemia, retinopathy, nephropathy, hypoglycemia, longer duration of diabetes, increased number of comorbidities, older age, and fewer years of formal education were found to be associated with MCI. The multivariable model indicated fewer years of formal education, previous CVD, hypertension, increased number of comorbidities, higher HbA1c and IL-1β levels, and lower adiponectin level. Elderly diabetic patients with MCI have higher levels of leptin and IL-1β and lower levels of adiponectin.
Kamogawa et al. (2010) nd Waragai et al. (2017)	517middle-aged-to-elderly community-dwelling persons.	Abdominal fat, adipose-derived hormones and mild cognitive impairment: the J-SHIPP study.	Mild cognitive impairment (MCI)	Serum, ELISA	In men, the abdominal subcutaneous fat area was significantly lower in participants with MCI than in those with normal cognitive function
Teixeira et al. (2013) and Sekiyama et al. (2014)	54 subjects with MCI and 43 controls	Decreased levels of circulating adiponectin in mild cognitive impairment and Alzheimer’s disease.	Mild cognitive impairment (MCI) and Alzheimer’s disease (AD)	Serum, ELISA	Serum levels of adiponectin were significantly lower in MCI and AD as compared to controls (*p* < 0.001). After controlling for age, educational level, and APOE genotype, adiponectin levels remained significantly reduced in these groups (*p* < 0.001). Circulating adiponectin levels did not predict cognitive decline in the elderly controls (i.e., progression from normal cognition to MCI) or progression to Alzheimer’s disease in subjects with MCI.
Letra et al. (2019a) and Gorska-Ciebiada et al. (2016)	Human, amnestic mild cognitive impairment (MCI, *n* = 71) and Alzheimer’s dementia (AD, *n* = 53)	Association between adipokines and biomarkers of Alzheimer’s disease: A cross-sectional study.	Alzheimer’s disease	serum and CSF, ELISA	Serum adiponectin was 33% higher in AD when compared to MCI patients. Adiponectin CSF levels, similar in both groups, were positively correlated with Aβ42 and cognitive function, though only in women. The area under the ROC curve was 0.673 (95% CI: 0.57–0.78) for serum adiponectin as predictor of dementia stage and the cutoff 10.85 μg/ml maximized the sum of specificity (87%) and sensitivity (44%).
Fujita et al. (2018) and Kamogawa et al. (2010)	20 male and 32 female, aged 60–93 years, mean 80.0	Increased adiponectin is associated with cerebral white matter lesions in the elderly with cognitive impairment	Alzheimer’s disease	Serum, ELISA	High serum adiponectin levels correlated with more severe WML (*p* = 0.013). Low BMI (*p* < 0.001), female sex (*p* = 0.025), and high WML scores (*p* = 0.039) were significant determinants of high serum adiponectin. HT (*p* = 0.032) and high adiponectin levels (*p* = 0.021) were independent risk factors for WML. Overall, we observed an association between serum adiponectin levels and WML severity in elderly people with cognitive decline.
Bednarska-Makaruk et al. (2017) and Teixeira et al. (2013)	205 patients with dementia [89 with Alzheimer’s disease (AD), 47 with vascular dementia (VaD), 69 with mixed dementia (MD)], 113 persons with mild cognitive impairment and in 107 controls	Association of adiponectin, leptin, and resistin with inflammatory markers and obesity in dementia.	Dementia.	Serum and CSF, ELISA	In all-cause dementia, adiponectin and resistin levels were significantly higher as compared to the controls; leptin levels did not show differences. Higher adiponectinlevels concerned AD and MD, whereas higher resistin concerned VaD and MD. After stratification by abdominal obesity, the differences in adiponectin levels remained significant in subjects without obesity. In all-cause dementia, negative correlation of adiponectin with obesity, glucose metabolism parameters, IL-6, and hsCRP and positive correlation with HDL-cholesterol were found. Positive correlation of resistin with age, IL-6, hsCRP, and chitotriosidase and negative correlation with HDL-cholesterol and paraoxonase 1 were stated.
Une et al. (2011) and Letra et al. (2019a)	Normal controls (*n* = 28), MCI (*n* = 18), and AD (*n* = 27) subjects	Adiponectin in plasma and cerebrospinal fluid in MCI and Alzheimer’s disease.	MCI and Alzheimer’s disease.	Serum and CSF, ELISA,	The levels of adiponectin in plasma and in CSF showed a positive correlation. Plasma adiponectin was significantly higher in MCI and AD compared to NC, whereas CSF adiponectin was significantly higher in MCI compared to NC.
Gilbert et al. (2018) and Fujita et al. (2018)	205 patients over 65 years of age	Association between peripheral leptin and adiponectin levels and cognitive decline in patients with neurocognitive disorders ≥65 years.	Neurocognitive disorders	Serum, ELISA	The mean BMI was significantly lower (by 2 kg/m^2^, *p* = 0.01) in patients with AD than in patients with either mild-NCD or vascular/mixed dementia. Leptin levels were significantly higher (*p* = 0.043) and adiponectin levels were significantly lower (*p* = 0.045) in patients with mild-NCD than in patients with major-NCD (AD or vascular/mixed dementia). However, the mixed model suggested no influence ofthe baseline levels of these two biomarkers on the course of cognitive decline.
Benavente et al. (2019) and Bednarska-Makaruk et al. (2017)	50% splits of TARCC’s data (Group 1 *n* = 1,691; Group 2 *n* = 1,690)	Serum adiponectin is related to dementia.	Alzheimer’s disease	MIMIC models	Serum APN was significantly related to δ scores (*r* = 0.10, *p* = 0.015). APN hadno significant effect on g’ (*r* = −.25, *p* = 0.66), nor did it have any independent direct effects on cognitive performance. These results were replicated across random subsets (ΔCHISQ = 2.8 (Scherer et al., 1995), *p* > 0.90).
Bossolasco et al. (2017) and Une et al. (2011)	88 samples in the whole cohort	Adiponectin levels in the serum and cerebrospinal fluid of amyotrophic lateral sclerosis patients: possible influence on neuroinflammation?	Cerebrovascular and neurodegenerative diseases	Serum and CSF, ELISA	In the whole ALS group, serum APN levels were not different when compared to the age- and sex-matched control group (CTR), but a gender-specific analysis enlightened a significant opposite APN trend between ALS males, characterized by lower values (ALS 9.8 ± 5.2 vs. CTR 15 ± 9.7 μg/ml), and ALS females, showing higher amounts (ALS 26.5 ± 11.6 vs. CTR 14.6 ± 5.2 μg/ml). This sex-linked difference was significantly enhanced in familial ALS cases (*p* ≤ 0.01). The APN levels in ALS cerebrospinal fluids wereunrelated toserum values and not linked to sex and/or familiarity of the disease. Finally, the screening of serum APN levels in patients affected by other neurological disorders revealed the highest serum values in FTD patients.
Kitagawa et al. (2016) and Gilbert et al. (2018)	466 patients (mean age 67.8 years, male 57%)	Serum high-molecular-weight adiponectin level and incident dementia in patients with vascular risk factors.	Dementia	Serum, ELISA	Serum HMW adiponectin level was 4.33 ± 2.95 μg/ml; the levels were lower in men than in women and negatively correlated with body mass index. During the follow-up period (median 6.9 years), 47 patients had incident dementia including Alzheimer’s disease dementia (Fillit et al., 2008), vascular dementia (Gustafson, 2010), mixed dementia (four), other dementia (three). Risks of dementia in patients with high vs. low HMW adiponectin levels were almost identical (*p* = 0.689). No association was found between adiponectin levels and Alzheimer’s disease dementia or vascular dementia in the whole group or among men and women separately.
Dukic et al. (2016) and Benavente et al. (2019)	235 participants	The role of human kallikrein 6, clusterin and adiponectin as potential blood biomarkers of dementia.	Dementia.	Serum, ELISA	Serum concentrations of KLK6 (*p* = 0.137), CLU (*p* = 0.178), and ADPN (*p* = 0.268) did not differ between AD, VAD, MCI and cognitively healthy control group of participants, whereas IL-6 was significantly higher in VAD patients than in AD, MCI, and CHP individuals (*p* = 0.014). There was no association between investigated biomarkers and clinical patient parameters.

First of all, the most important one is that individual diagnostic accuracy varies in different study teams. Classification of patients in cohorts depends on the accurate diagnostic process. Using diagnostic tools [such as Mini Mental State Examination (MMSE) and Montreal Cognitive Assessment (MoCA)] or exclusively relying on them, ignoring the limitation of examinations may influence the result (Chapman et al., 2016). Second, lack of detailed treatment data for patients enrolled, such as drugs and other treatments. Certain special drugs such as acetylcholinesterase inhibitors (AChEIs), statins, and thiazolidinediones all might affect the circulating levels of APN (Montecucco and Mach, 2009). Third, the nonuniformity in basic characteristics of study design and studied populations or lack of adjustments to confounding factors including vascular risk factors may also explain the inconsistency of different experimental results. Fourth, few studies have distinguished different proportions of circulating APN isomers, which are the decisive isomers, hexamers, trimers, or HMW. As mentioned above, different forms determine their different biological activities. In related diseases, HMW APN or HMW APN/total APN may be a more effective indicator of insulin sensitivity (Aso et al., 2006). Lastly, high circulating APN levels may result in subsequent resistance to APN in a manner similar to IR. Studies have demonstrated that high circulating APN levels are related to inverse outcome, defined as APN resistance (Van Berendoncks et al., 2010; Sente et al., 2016). Thus, APN may show dual effects in the pathological process of dementia.

## Conclusion and Future Perspective

Researches about dementia are rapidly increasing. Advances in basic science and clinic studies in molecular and pathological mechanism have provided unprecedented possibilities for novel therapeutic strategy. The association between APN and AD or VD, whether direct or indirect, positive or negative, in risk factors or in pathological mechanism, central or peripheral, has been deeply evaluated and elaborated in numerous studies. Overall, it is significantly associated with risk factors of dementia such as obesity, T2DM, hyperlipidemia, atherosclerosis, and other vascular factors. APN has multiple effects on the pathological process of dementia. Positive effects are involved in anti-inflammation, reduced IR, anti-atherosclerosis, and regulation of energy metabolism, such as glycogen and lipids. Insulin sensitivity and neuroinflammatory responses are key cellular mechanisms involved in age-related cardiovascular disease, metabolic disease, cerebrovascular dysfunction, and cognitive decline. APN is highly correlated with insulin sensitivity and inflammatory response. Many studies have shown that elevated levels of APN can improve damaged insulin signaling; inhibit neuroinflammation, oxidative stress, nitrosative stress, etc.; and affect cerebral blood vessels. However, its potential mechanism is still not well explored and even controversial. Further studies are required to illustrate the exact functions and receptor-dependent or -independent downstream pathways of each isoform activation, and how APN’s peripheral concentration can modulate its central effect. This will help us enhance congruency of the results and further facilitate the search for the possible role and pathophysiological mechanism of APN in the onset and development of dementia, especially its actions on the hippocampus and cerebral cortex. Finally, identifying and using these potential relationships, and thoroughly understanding APN’s physiology, may help us seek out a multi-target cocktail therapy for individuals with cognitive impairment disease, or find a novel way to delay or block the process of dementia in the early stage. Is there an individual treatment that can take into account of the different physiological characteristics, genotypes, comorbidities, or even individual biomarker levels of dementia patients to achieve a cure? Maybe it’s no longer a dream in the coming future.

## Author Contributions

YZ determined the structure of the review. RC selected the references and contributed to the writing. YS contributed to the revision and finalization of the article.

## Conflict of Interest

The authors declare that the research was conducted in the absence of any commercial or financial relationships that could be construed as a potential conflict of interest.
